# Anti-osteoporotic treatments in the era of non-alcoholic fatty liver disease: friend or foe

**DOI:** 10.3389/fendo.2024.1344376

**Published:** 2024-03-08

**Authors:** Maria Eleni Chondrogianni, Ioannis Kyrou, Theodoros Androutsakos, Christina-Maria Flessa, Evangelos Menenakos, Kamaljit Kaur Chatha, Yekaterina Aranan, Athanasios G. Papavassiliou, Eva Kassi, Harpal S. Randeva

**Affiliations:** ^1^ Department of Biological Chemistry, Medical School, National and Kapodistrian University of Athens, Athens, Greece; ^2^ Endocrine Unit, 1st Department of Propaupedic Internal Medicine, Laiko Hospital, National and Kapodistrian University of Athens, Athens, Greece; ^3^ Laboratory of Dietetics and Quality of Life, Department of Food Science and Human Nutrition, School of Food and Nutritional Sciences, Agricultural University of Athens, Athens, Greece; ^4^ Warwickshire Institute for the Study of Diabetes, Endocrinology and Metabolism (WISDEM), University Hospitals Coventry and Warwickshire NHS Trust, Coventry, United Kingdom; ^5^ Institute for Cardiometabolic Medicine, University Hospitals Coventry and Warwickshire NHS Trust, Coventry, United Kingdom; ^6^ Warwick Medical School, University of Warwick, Coventry, United Kingdom; ^7^ Centre for Health & Life Sciences, Coventry University, Coventry, United Kingdom; ^8^ Aston Medical School, College of Health and Life Sciences, Aston University, Birmingham, United Kingdom; ^9^ College of Health, Psychology and Social Care, University of Derby, Derby, United Kingdom; ^10^ Department of Pathophysiology, Medical School, National and Kapodistrian University of Athens, Athens, Greece; ^11^ 5th Surgical Clinic, Department of Surgery, ‘Evgenidion Hospital’, National and Kapodistrian University of Athens Medical School, Athens, Greece; ^12^ Department of Biochemistry and Immunology, University Hospitals Coventry and Warwickshire NHS Trust, Coventry, United Kingdom

**Keywords:** anti-osteoporotic drugs, non-alcoholic fatty liver disease, denosumab, romosozumab, bisphosphonates, calcitonin, selective estrogen receptor modulators, PTH

## Abstract

Over the last years non-alcoholic fatty liver disease (NAFLD) has grown into the most common chronic liver disease globally, affecting 17-38% of the general population and 50-75% of patients with obesity and/or type 2 diabetes mellitus (T2DM). NAFLD encompasses a spectrum of chronic liver diseases, ranging from simple steatosis (non-alcoholic fatty liver, NAFL) and non-alcoholic steatohepatitis (NASH; or metabolic dysfunction-associated steatohepatitis, MASH) to fibrosis and cirrhosis with liver failure or/and hepatocellular carcinoma. Due to its increasing prevalence and associated morbidity and mortality, the disease-related and broader socioeconomic burden of NAFLD is substantial. Of note, currently there is no globally approved pharmacotherapy for NAFLD. Similar to NAFLD, osteoporosis constitutes also a silent disease, until an osteoporotic fracture occurs, which poses a markedly significant disease and socioeconomic burden. Increasing emerging data have recently highlighted links between NAFLD and osteoporosis, linking the pathogenesis of NAFLD with the process of bone remodeling. However, clinical studies are still limited demonstrating this associative relationship, while more evidence is needed towards discovering potential causative links. Since these two chronic diseases frequently co-exist, there are data suggesting that anti-osteoporosis treatments may affect NAFLD progression by impacting on its pathogenetic mechanisms. In the present review, we present on overview of the current understanding of the liver-bone cross talk and summarize the experimental and clinical evidence correlating NAFLD and osteoporosis, focusing on the possible effects of anti-osteoporotic drugs on NAFLD.

## 1 Introduction

Non-alcoholic fatty liver disease (NAFLD; or metabolic dysfunction-associated fatty liver disease, MAFLD; or metabolic dysfunction-associated steatotic liver disease, MASLD) is one of the most common causes of chronic liver disease worldwide. Indeed, over the past couple of decades NAFLD has grown into the most common chronic liver disease, with prevalence of 17-38% in the general population (1), and 50-75% in patients with obesity and/or type 2 diabetes mellitus (T2DM) (2, 3). NAFLD is determined as steatosis affecting 5% of the liver volume or weight (accumulation of fat in more than 5% of hepatocytes) (4), and encompasses a spectrum of liver diseases, ranging from simple steatosis (non-alcoholic fatty liver, NAFL) and non-alcoholic steatohepatitis (NASH or metabolic dysfunction-associated steatohepatitis, MASH) to fibrosis and cirrhosis with liver failure or/and hepatocellular carcinoma. Notably, based on recent estimates, cirrhosis due to NAFLD is expected to be the leading cause of liver transplantation in the US by 2030 (5). Additionally, the economic burden of NAFLD/NASH on health systems is enormous, while that there is currently no globally approved treatment specifically for NAFLD/NASH (6).

Currently, NAFLD is diagnosed by detecting steatosis (either by imaging or histologically) and by excluding other causes of liver disease, including exclusion of alcoholic liver disease (ALD) which has similar pathologic spectra with NAFLD (in NAFLD the daily alcohol consumption should not exceed 20 g in women or 30 g in men) (7). For the diagnosis, as well as its staging, of NASH and cirrhosis, liver biopsy remains the ‘gold standard’, which, however, also has limitations since it is an invasive method with possible complications, sampling errors and high cost (8, 9). Of note the renaming of NAFLD as MAFLD or MASLD has recently been proposed, with new proposed diagnostic criteria, namely hepatic steatosis based on histological (biopsy), imaging or biochemical confirmation along with one of the following: (a) overweight/obesity; (b) T2DM; or (c) metabolic dysfunction as indicated by 2 of the following: increased waist circumference, hypertension, elevated triglycerides, low HDL, prediabetes (IGT, IFG), HOMA index>2.5, elevated CRP, which rather put a positive diagnosis and not an exclusion of other causes of liver disease (10).

Osteoporosis is also a chronic disease characterized by decreased bone density and a disruption of the bone’s architectural structure, resulting in bone fragility and increased fracture risk (11). Osteoporosis is particularly prevalent in postmenopausal women, where the noted estrogen decrease leads to an increase of the activity of the osteoclasts, increasing their responsiveness to RANKL which binds to RANK on the osteoclast membrane, and resulting in the differentiation of osteoclast precursors into mature osteoclasts (12). The drugs used for the treatment of osteoporosis are antiresorptives (e.g. bisphosphonates, denosumab and raloxifene), bone anabolic agents (e.g. teriparatide and romosozumab) and calcitonin (13). Similar to NAFLD, osteoporosis progresses as a silent disease until an osteoporotic fracture occurs, which also poses a very significant disease-related and socio-economic burden (6).

Recent data have been highlighting potential links between NAFLD and osteoporosis, linking the pathogenesis of NAFLD to the process of bone remodeling. In this context, it is considered that chronic low-grade inflammation plays a crucial role in the pathogenesis of both diseases (14, 15). However, this field is still open and more evidence is needed towards understanding the potential common pathogenetic mechanism(s) and the correlations/links between NAFLD and osteoporosis.

In the present review, we present an overview of the current understanding of the liver-bone cross talk and summarize the experimental and clinical evidence correlating NAFLD and osteoporosis. Since these two diseases frequently co-exist, medications for the treatment of osteoporosis may affect NAFLD progression by impacting on underlying pathogenetic mechanisms/links. As such, herein, we also focus on insights into the possible effects of anti-osteoporotic drugs on NAFLD.

## 2 Pathogenetic mechanisms linking NAFLD and osteoporosis

Potential common pathogenetic mechanisms linking NAFLD and osteoporosis have been recently described. The pathogenesis of NAFLD was initially described with the ‘two-hit’ hypothesis, with steatosis, i.e. the accumulation of triglycerides (TAGs) in the liver, representing the ‘first hit’ and triggering the expression of pro-inflammatory cytokines (e.g. NF-a, IL-6) which was described as the ‘second’ hit (16). The latter results to the activation of pro-inflammatory pathways and potential fibrogenesis in the liver. However, the spectrum of mechanisms implicated in NAFLD pathogenesis appears to be much more complex, and, thus, has been more recently described with the “multiple-hit hypothesis” which includes multiple genetic and environmental factors that may result to obesity, insulin resistance, gut microbiome alterations, adipose tissue dysfunction and liver fat accumulation with or without hepatic inflammation (17). For example, hepatic mitochondrial dysfunction leads to endoplasmic reticulum (ER) stress, oxidative stress and the production of reactive oxygen species (ROS), while autophagy and apoptosis also play crucial role in NAFLD (18–20). Of note, even certain gut microbiome modifications appear to trigger the expression of pro-inflammatory cytokines (e.g. IL-6, TNF-α), whilst all the aforementioned mechanisms combined with genetic factors and epigenetic alterations may lead to chronic liver inflammation (17).

In this context, focus has been placed not only on traditional cytokines (e.g. IL-6, TNF-α), but also on additional factors implicated in these pathways such as adipokines (21, 22). Among these, adiponectin is the most abundant and is secreted predominantly by the white adipose tissue. Notably, adiponectin has anti-inflammatory and anti-atherogenic effects, and plays an important role in lipid and glucose metabolism by increasing insulin sensitivity, promoting the oxidation of free fatty acids, decreasing *de novo* synthesis/accumulation of lipids and protecting hepatic cells from apoptosis (23, 24). The effects of adiponectin on the liver are mediated by its receptors (AdipoR1, AdipoR2), interacting with the adaptor protein phosphotyrosine interaction (APPL1). Indeed, AdipoR1 activates the AMP-activated protein kinase (AMPK) and AdipoR2 the peroxisome proliferator–activated receptor-alpha (PPAR-a) signaling, and, thus, through these pathways adiponectin acts against hepatic lipid accumulation and regulates glucose homeostasis. Moreover, through the blockage of nuclear factor kappa (NF-κβ), adiponectin reduces inflammation. Notably, a diet rich in saturated and trans-fats, which directly induces significant hepatic fatty infiltration, has been shown to also reduce the circulating levels of adiponectin (25, 26). Furthermore, data show that adiponectin impedes hepatic fibrosis, by inhibiting platelet-derived growth factor (PDGF) stimulation and downregulating the transforming growth factor beta 1 (TGF-β1) (26), whilst as NAFLD progresses, adiponectin levels appear to decline (27). Interestingly, expression of adiponectin receptors has also been found in bone cells, both osteoblasts and osteoclasts (28). In addition, data from *in vitro* experiments and animal studies support an osteogenic role for adiponectin, by promoting osteoblastogenesis and limiting osteoclastogenesis (29). Accordingly, since NAFLD is associated with decreased adiponectin levels, the adiponectin downstream signaling pathways may favor osteoclast function and bone loss in patients with NAFLD. However, it should be noted that human data do not consistently point towards a favorable effect of adiponectin on bone biology (28, 29). Bacchetta et al. showed a negative association between bone mineral density (BMD) and adiponectin in patients with chronic kidney disease (30), whilst Jürimäe et al. demonstrated a negative correlation between adiponectin and BMD in a group of middle-aged premenopausal women (31). Moreover, a recent case-control study, including 210 postmenopausal women, showed an inverse relationship between serum adiponectin levels and T-score in women with osteoporosis and osteopenia (32). Finally a prospective study by Barbour et al., showed that high adiponectin levels were correlated with a higher fracture risk in men, but not in women (33).

Recent research focus has also been placed on osteocalcin (OC) which is secreted by osteoblasts and constitutes the most abundant non-collagen protein in bone (34). In its uncarboxylated form, OC exerts effects on bone by binding calcium (35), whilst it also plays a role on the pancreas-liver crosstalk and metabolism by promoting directly insulin expression in the pancreas and by increasing GLP-1 and adiponectin expression in adipocytes (36). Conversely, OC expression in osteoblasts is promoted by insulin and adiponectin (37). Several studies have demonstrated an inverse association between NAFLD and serum OC levels, with Yilmaz et al. showing that patients with NAFLD and increased hepatocyte ballooning degree had lower OC levels (38). Furthermore, Yang et al. demonstrated that Korean men with NAFLD had lower BMD and OC levels compared to those without (39), whilst Luo et al. showed that among postmenopausal Chinese women with normal blood glucose levels those with NAFLD had lower OC levels (40). Finally, Fang et al. also revealed that lower serum OC levels was an independent risk factor for NAFLD and progression to NASH (41). Interestingly, the hepatic inflammation observed during the progression of NAFLD due to lipotoxicity and the production of pro-inflammatory (e.g., TNF-α, IL-1, IL-6, IL-17) and prothrombotic factors appears to affect both the pathogenesis of NAFLD and bone tissue metabolism (14). RANKL, RANK and osteoprotegerin (OPG) are osteokines which are expressed in bone cells and regulate bone remodeling (42). The OPG/RANKL balance is highly important for the maintenance of bone health, with denosumab, a RANKL-binding monoclonal antibody, being approved as an anti-osteoporotic treatment (42). Of note, upregulation of the RANK/RANKL pathway induces the expression of pro-inflammatory cytokines, such as IL-1, IL-6, and TNF-α, which, in turn, promote osteoclast activation and bone resorption (43). RANKL binds to RANK on the osteoclast membrane, resulting in the differentiation of osteoclast precursors into mature osteoclasts (12), whilst OPG diminishes osteoclastogenesis and, thus, bone loss by binding to RANKL, preventing the RANK-RANKL activation of osteoclasts. Potential associations between RANKL, OPG and NAFLD have been investigated, with experimental data showing that the hepatic expression of RANKL may be elevated in patients with NAFLD (44). Furthermore, a study by Amrousy et al. in children with obesity and NAFLD showed that these children had both higher TNF-a and IL-6 levels and lower OC, OPG and adiponectin levels compared to the study controls (45). Moreover, Mantovani et al., trying to access bone turnover markers in postmenopausal T2DM patients with and without NAFLD (10 patients with NAFLD and fibrosis, 52 with NAFLD and without fibrosis, and 15 without NAFLD), found that RANKL levels gradually diminished from the study patients without NAFLD, to those with steatosis and then to those with steatosis and fibrosis, while sclerostin levels were higher in patients with NAFLD (46). Another study by Niksersht et al. in a sample of 57 men with NAFLD and 25 controls demonstrated that patients with NAFLD had lower levels of RANKL and OPG compared to controls, whilst OPG and RANKL gene expression was also reduced, suggesting a potential role in NAFLD pathogenesis (47). Similarly, Hadinia et al. showed that patients with NAFLD exhibited lower plasma RANK levels compared to controls, with diminished mRNA RANK levels (48). A previous study by Yilmaz et al. had also demonstrated that serum OPG levels were lower in patients with NASH compared to controls, suggesting that OPG could be used as a biomarker for NASH (49). A case-control study Niu et al. including T2DM patients with NAFLD (N=367) and without (N=379) NAFLD showed that OPG levels were lower in those with NAFLD (50). Interestingly, Erol et al., trying to investigate whether there is a correlation between OPG and insulin resistance in children with obesity, found that OPG levels were lower in such children, but failed to detect a difference in OPG concentrations between children with both obesity and NAFLD compared to those with obesity without NAFLD (51). Finally, Ayaz et al. have shown that serum OPG levels and carotid intima media thickness (CIMT) were higher in patients with NAFLD, with a positive association between OPG and CIMT in these patients (52). It appears that most of the studies point towards a positive correlation of serum RANKL with NAFLD, while serum OPG decreases with disease severity. Though, high serum OPG and low serum RANKL levels have also been reported in patients with advanced NAFLD-related fibrosis (46). It is difficult to explain this discrepancy, however, it should be noted that the elevation of circulating OPG levels and the decreased RANKL levels could represent a compensatory mechanism to limit the liver damage during the progress of NAFLD to fibrosis.

Of note, OPG and other molecules involved in bone metabolism, such as osteopontin (OPN) have also been associated with the progression of hepatic fatty infiltration to fibrosis (14). Indeed, focus is now placed on OPG which is a member of the TNF-a receptor family and acts as a cytokine by preventing RANK from binding to RANKL (53). In this context, Yang et al., in an attempt to suggest OPG as a noninvasive biomarker for NASH diagnosis and NAFLD progression, found that serum OPG was lower in NASH patients compared to normal controls (54). Similarly, OPN is a glycoprotein that plays a role in bone remodeling, bone matrix mineralization, bone remodeling, cell chemotaxis and cell survival and apoptosis (55). Bertola et al. studied the hepatic expression of OPN and its surface receptor CD44, in patients with obesity, showing that hepatic OPN levels were higher in those with severe steatosis and insulin resistance, suggesting their local implication in the hepatic injury progression (56). Moreover, Gómez-Santos et al. showed that OPN levels are higher in older patients, whilst this finding did not apply to patients with NAFLD where higher OPN levels were noted in younger patients. By also studying OPN deficient mice during aging, this study also showed that in older mice decreased OPN levels resulted in augmented senescence, ER stress, hepatic steatosis, and inflammation (57).

Insulin-like growth factor-1 (IGF-1) is mainly secreted by the liver while is also locally produced in small amounts by bones, affecting positively bone remodeling (58). Yao et al. in their metanalysis demonstrated that IGF-1 levels were decreased in patients with NAFLD compared to healthy controls (59). Moreover, Dichtel et al. showed that decreased IGF-1 levels were correlated with higher histological severity of NAFLD (60). Decreased IGF-1 have been reported in both patients with osteoporosis and NAFLD, indicating the important role of IGF-1 in the liver-bone axis (58).

To this end, Wang et al. investigated the role of IGF-1 in the progression of both NAFLD and osteoporosis (61). Using 48 female mice divided into two groups, WT and fed with high fat diet, they showed that bone loss, deterioration of bone microarchitecture and NAFLD were progressing in parallel. They demonstrated that changes of the TNF‐α, IL‐6, as well as IGF‐ 1 and IGFBP‐1 levels appear to play crucial roles in the different stages of NAFLD in HFD-fed mice. In particular, they showed that in 24 weeks the levels of TNF-α and IL-6 were higher in mice fed with HFD compared to controls leading to changes in the OPG/RANK/RANKL pathway. They concluded that changes in bone microstructure and BMD regarding the ‘second hit’ were due to higher levels of TNF-a and IL-6. They also showed that, in 32 weeks, IGF-1 was lower in mice fed with HFD resulting to reduced osteoblast activity, justifying bone changes in the progressive stage of NAFLD (61).In addition to the aforementioned factors, vitamin D deficiency, which has known effects on bone metabolism, seems to also influence NAFLD progression by inducing pro-inflammatory processes and oxidative stress, as well as stimulating the proliferation of stellate cells and the production of pro-fibrotic factors (e.g. PDGF and TGFβ) (14). Indeed, The role of vitamin D deficiency in the progression of NAFLD has been demonstrated in animal models (14), whilst clinical data, such as those from Wang et al., also suggest that 25(OH)-vitamin D levels are lower in patients with NAFLD (62).

Advanced glycation end-products (AGEs), molecules deriving from the glycation of proteins or lipids, seem to play a role in both the pathogenesis of NAFLD and osteoporosis (63, 64). Asadipooya et al. in their review provided a thorough description of how AGEs, through their receptors (RAGE), provoke inflammation, cellular proliferation, and increased oxidative stress that lead to the progression of steatosis to NASH and fibrosis, while vice versa oxidative stress and inflammation trigger the AGEs production (63). Of note, AGEs are also involved in bone metabolism. At low concentrations, AGEs promote osteoblastic activity, but at higher concentrations impair mineralization, induce osteoclastogenesis by upregulating RANKL, restrain osteoblasts’ growth, inhibit their differentiation and promote their apoptosis (64). Thus, targeting AGE-RAGE signaling appear to be very promising in preventing the progression of both NAFLD and bone loss.

Finally, emerging data also suggest that the Wnt signaling pathway may contribute to the liver-bone crosstalk. The role of the Wnt signaling pathway in osteogenesis is well-described, with Wnt-derived proteins diminishing apoptosis in osteoblast precursor cells and promoting osteoblast differentiation (65). Similarly, the role of Wnt/beta catenin signaling - where the binding of a Wnt ligand with a surface receptor (Fzd) and a co-receptor (LRP5/6) is responsible for the stabilization of beta/catenin, its nuclear translocation and Wnt target gene expression - in NAFLD development has been described, as recently reviewed in detail by Harini et al. (66). In this context, the role of the canonical and non- canonical Wnt pathway is considered crucial in the development of NAFLD, with the latter being promoted or suppressed according to Wnt5a binding, whilst NAFLD can induced by the inhibition of the canonical pathway. As such, it is noteworthy that mutations in the Wnt co-receptor low density lipoprotein (LDL) receptor-related protein 6 (LRP6) can provoke NAFLD (66), with Liu et al. demonstrating that LRP6^+/-^ mice were protected against insulin resistance and obesity (67).

## 3 Studies on NAFLD and osteoporosis links

A number of mostly cross-sectional studies have examined the interrelation between NAFLD and BMD, as presented in **Table 1**. Of these, several demonstrated that patients with NAFLD had higher risk of osteoporosis. For example, Chen et al. have shown that there is a decrease in the rate of bone production and an increase in the rate of bone resorption in elderly patients with NAFLD relative to individuals without NAFLD (78). Similarly, a recent study by Lee et al. in men older than 50 years showed a higher 10-year probability of a major osteoporotic fracture in those with NAFLD compared to those without, while this association was more pronounced in those with sarcopenia (82). Contrary, there are also studies demonstrating either no correlation or a positive correlation between NAFLD and BMD (**Table 1**). However, as summarized in **Table 1**, several limitations make the findings of these studies questionable, particularly since no liver biopsies were performed to conclusively diagnose/stage NAFLD and fractures were self-reported, whilst various confounding factors (e.g. vitamin D levels, metabolic bone markers, other medications) were not accessed/included in the analysis. Meta-analysis data correlating NAFLD and osteoporosis have been presented by Su et al. which showed that NAFLD is associated with decreased BMD and higher risk of osteoporosis or osteoporotic fractures, with male sex potentially being a risk factor for decreased BMD in adults with NAFLD, whilst ethnic disparities appear to be also present between non-Asian and Asian populations regarding both BMD and osteoporotic fractures (87). Moreover, the systematic review and meta-analysis by Pan et al. which included seven eligible studies showed a significant association between NAFLD and the prevalence and risk of osteoporosis or osteoporotic fractures in both men and women (88).

**Table 1:** Selected studies (in chronological order) on the association between non-alcoholic fatty liver disease (NAFLD) and osteoporosis/bone mineral density (BMD) in humans.

	Study design	Origin	Study population	Methods	Outcome	Limitations
Li et al., 2012 (68)	Cross-sectional	China	7797 participants over 40 years old, (2441 men and 5356 women), 2352 with NAFLD	Questionnaire	Association between NAFLD and osteoporotic fractures in men but not in women	1. no causal inference due to cross-sectional design; 2. self-report, thus asymptomatic fractures could not be reported; 3. no biopsy for the diagnosis of NAFLD; 4. confounding factors, such as dietary calcium intake or serum 25-hydroxyvitamin D that could not be ruled out
Moon et al., 2012 (69)	Cross-sectional	South Korea	481 adult women (216 premenopausal and 265 postmenopausal)	DEXA lumbar BMD	Postmenopausal women without NAFLD had higher lumbar BMD compering to those with NAFLD, therefore there was no difference found in premenopausal women	1. no causal inference due to cross-sectional design; 2. waist circumference measurement to define metabolic syndrome was not available in all patients; 3. no biopsy for the diagnosis of NAFLD
Purnak et al., 2012 (70)	Cross-sectional	Turkey	102 adults patients with NAFLD and 54 healthy controls	DEXA	No correlation between NAFLD and lower BMD. Subgroup analysis demonstrated that women with higher ALT levels had a lower BMD and higher hs-CRP levels	1. no biopsy for the distinguish of NAFLD; 2. conflicting results
Cui et al., 2013 (71)	Cross-sectional	China	224 adults; 99 men (46 with NAFLD, 53 without NAFLD), and 125 women (73 with NAFLD 52 without NAFLD)	DEXA	Men with NAFLD had significantly lower TH and FN BMD and women with NAFLD had Lower right TH BMD	1. cross-sectional design; 2. confounding factors; 3. no liver biopsies
Xia et al., 2016 (72)	Cross-sectional	China	1659 adults (755 men; 904 women)	DEXA	LFC and ALT were inversely associated with lower BMD regarding multiple skeletal sites in middle-aged men, but no association was found in postmenopausal women	1. cross-sectional design; 2. no liver biopsies; 3. sex steroid hormones were not evaluated
Lee et al., 2016 (73)	Cross-sectional	South Korea	6634 adults (3306 men: 1288 with NAFLD; 2018 without NAFLD; 3328 women: 1217 with NAFLD; 2112 without NAFLD)	DEXA	FN BMD was negatively correlated with NAFLD in men and LS BMD was positively correlated with NAFLD in women	1. cross-sectional design; 2. no liver biopsies; 3. confounding factors
Yang et al., 2016 (39)	Cross-sectional	South Korea	859 adult men (249 with and 610 without NAFLD)	DEXA	NAFLD was negatively associated with right TH BMD and serum osteocalcin in Korean men.	1. cross-sectional design; 2. only men; 3. no biopsies; 4. confounding factors
Kim et al., 2017 (74)	Cross-sectional	South Korea	231 adults (160 women and 71 men); 129 with NAFLD	DEXA and transient elastography	Correlation between significant liver fibrosis and lower BMD among patients with NAFLD, using TE	1. cross-sectional deign; 2. no liver biopsies; 3. difficulties in the interpretation of elastography; 4. no bone turnover markers; 5. use of hormonal replacement therapy, HOMA-IR index and CRP levels were not accessed
Ahn et al., 2018 (75)	Cross-sectional	South Korea	4264 adults (1908 men 2356 women)	DEXA and FLI	Correlation between high FLI with lower BMD in men (TH, FM and whole body BMD)	1.cross-sectional design; 2. FLI, no biopsies where used; 3. FLI index differentiation between races; 4. no relationship between FLI and osteoporotic fractures was found because of the small number of fractures among patients in the study; 5. the effect of diabetes and anti-diabetic drugs on NAFLD was not evaluated.
Chen et al., 2018 (76)	Cross-sectional	China	938 postmenopausal women (365 with NAFLD, of those 132 with moderate/severe NAFLD)	DEXA	Moderate/severe NAFLD was independently correlated with osteoporosis and not mild, MetS was found to be an independent factor for osteoporosis combined addictive effect of moderate and severe NAFLD and MetS on osteoporosis	1. cross-sectional design; 2. retrospective study; 3. ultrasound or the diagnosis of NAFLD; 4. no metabolic markers; 5. only one center
Wang et al., 2018 (77)	Cross-sectional	China	2659 adults (950 men and 1709 women) of these 2045 with NAFLD	Ultrasound, questionnaire	NAFLD was correlated with the risk of osteoporotic fractures in men over 55 years old, but not in women. NAFLD was correlated with osteoporotic fractures in men without dyslipidemia	1. cross-sectional design; 2. recall bias; 3. self-reported fractures-missing vertebral; 4. confounding factors
Chen et al., 2018 (78)	Retrospective cohort study	China	4318 adults with NAFLD and 17272 without		Association between NAFLD and increased risk of new onset osteoporosis	1.confounding factors; 2. delay of diagnosis
Umehara, 2018 (79)	Cross-sectional	USA	6089 adults (1690 with NAFLD and 4399 without NAFLD)	DEXA	NAFLD was not significantly associated with BMD. NAFLD with higher ALT was negatively correlated with FN BMD	1. cross-sectional design; 2. confounding factors; 3. no fractures report; 4. ALT does not directly access the severity of NAFLD
Sung et al., 2020 (80)	Retrospective cohort study	South Korea	4536 adults (1006 men: 434 with NAFLD and 572 without NAFLD; 3530 women: 446 with NAFLD and 3084 without NAFLD)	DEXA	NAFLD was correlated with lower risk of BMD decrease in women	1. no biopsies, 2. young sample, 3. no bone metabolic markers
Ciardullo et al., 2021 (81)	Cross-sectional	USA	1784 adults (925 men and 859 women, 488 men and 391 women with liver steatosis and 126 men and 74 women with liver fibrosis)	DEXA, TE	No association between hepatic steatosis and hepatic fibrosis with osteoporosis	1. cross-sectional design; 2. no liver biopsies; 3. fracture risk was not accessed
Lee et al., 2021 (82)	Cross-sectional	Korea	2525 adults (FLI defined: 233 with NAFLD, 279 with NAFLD and fibrosis, CNS defined: 544 with NAFLD 614 with NAFLD and fibrosis)	Frax score	Association between NAFLD and a higher 10-year probability of major osteoporotic fracture in men >50, while this association was more pronounced in those with sarcopenia	1. cross-sectional design; 2. underestimated FRAX score due to missing data, 3. no biopsies
Xie et al., 2022 (83)	Cross-sectional	China	1980 adults (281 with NAFLD, 489 with severe steatosis)	DEXA Fibroscan	Negative correlation between NAFLD and BMD in persons aged 20 to 59 on subgroup analysis. A U-shaped relationship was found in black participants. In people aged 40-49 years, a positive relationship was found between BMD and advanced fibrosis and cirrhosis	1. cross-sectional design; 2. diagnosis with elastography; 3. missing data regarding medication, history of fracture; 4. no T scores and Z scores were reported
Yu et al., 2022 (84)	Cross-sectional	China	1243 diabetic patients (760 with NAFLD and 483 without NAFLD)	DEXA, ultrasound, FIB 4, NFS	Association between NAFLD (high risk for liver fibrosis) and osteoporosis in postmenopausal women with diabetes mellitus, but not in men	1. cross-sectional design; 2. no liver biopsies; 3. only middle and high risk according to NFS
Hassan et al., 2023 (85)	Cross-sectional	Egypt	100 adults (50 with NAFLD)	DEXA lumbar BMD	Association between NAFLD and lower BMD	1. small sample; 2. ultrasound for the diagnosis of NAFLD/no liver biopsy, 3. minimal steatosis could not be diagnosed; 4. the role of diabetes was not accessed; 5. only LS BMD was measured
Liu et al., 2023 (86)	Cross-sectional	USA	817 (381 with NAFLD 436 without NAFLD)	DEXA femoral BMD	NAFLD was correlated with higher BMD and lower risk of osteoporosis	1. cross-sectional study; 2. Possible ethnic disparities; 3. Questionnaires/recall bias; 4. No liver biopsies; 5. LS BMD was not accessed

ALT, alanine transaminase; BMD, bone mineral density; CRP, C-reactive protein; DEXA, dual x-ray absorptiometry; FLI, fatty liver index; FN, femoral neck; hs:high-sensitivity; HOMA-IR, homeostatic model assessment for insulin resistance; LS, lumbar spine; LFC, liver fat context; MetS, metabolic syndrome; NAFLD, non-alcoholic fatty liver disease; NFS, nafld fibrosis score; TE, transient elastography; TH, total hip.

## 4 Anti-osteoporotic treatments and NAFLD

**Figure 1:**
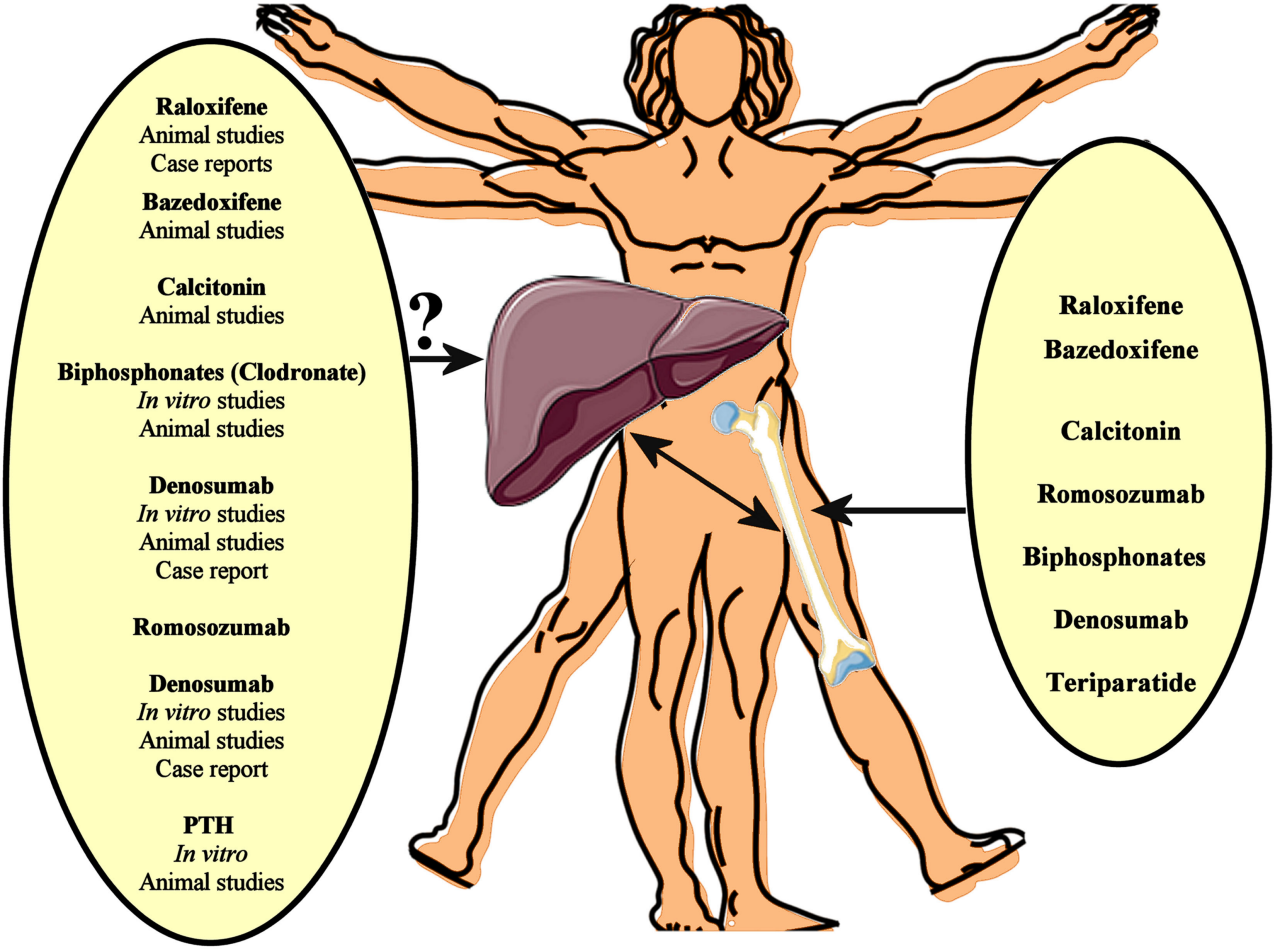
Several pharmacotherapies, including denosumab, bisphosphonates, teriparatide, raloxifene, calcitonin, and romosozumab, have well-established efficacy in the treatment of osteoporosis, reducing the risk of osteoporotic fractures. Given that osteoporosis and NAFLD frequently co-exist, such medications against osteoporosis may affect NAFLD progression by impacting on pathogenetic mechanisms/pathways shared by both these chronic diseases. *In vitro* data, animal studies and case reports support a beneficial effect of anti-osteoporotic drugs on the NAFLD/NASH progression. However, interventional studies could finally evaluate the potential impact of these anti-osteoporotic drugs on NAFLD.

Several pharmacotherapies, including denosumab, bisphosphonates, teriparatide, raloxifene, calcitonin, and romosozumab, have well-established efficacy in the treatment of osteoporosis, reducing the risk of osteoporotic fractures (89). Given that osteoporosis and NAFLD frequently co-exist, particularly in older adults, such medications against osteoporosis may affect NAFLD progression by impacting on pathogenetic mechanisms/pathways shared by both these chronic diseases (**Figure 1**).

### 4.1 Bisphosphonates and NAFLD

Bisphosphonates are a class of anti-osteoclastic drugs which are widely used as a pharmaceutical treatment for osteoporosis, constituting first-line therapeutic choices for osteoporosis. These have a structure like pyrophosphate and act by inhibiting bone resorption and remaining on the bone surface (90). Bisphosphonates are divided in nitrogen-containing and non-nitrogen-containing agents (91), and can be used continuously for 3-5 years. However, the long use of bisphosphonates may have side effects, such as atypical femoral fractures and jaw osteonecrosis (92). To date, no experimental or clinical study has showed that the nitrogen bisphosphonates may impact on NAFLD. However, there are experimental data from Hasuzawa et al. in a NASH mouse model induced by a methionine and choline deficient diet which show that clodronate (a non-nitrogen bisphosphonate which acts as a potent and selective inhibitor of the vesicular nucleotide transporter, VNUT) may improve NASH and diminish hepatic inflammation, steatosis, and fibrosis (93). *In vitro* experiments also showed that clodronate reduced hepatic neutrophil infiltration, hepatocyte apoptosis, and cytokine production, suggesting that VNUT-dependent vesicular ATP release plays a role in aggravating hepatic steatosis (93).

### 4.2 Selective estrogen receptor modulators and NAFLD

Selective estrogen receptor modulators (SERMS) act as estrogen agonists in the bone tissue, inhibiting the osteoclast activity, and as estrogen antagonists in breast and uterine tissues, thus exerting anti-osteoporotic effects without increasing the risk of breast cancer, as estrogen replacement therapy does; although, they increase the risk for thrombosis and pulmonary emboli (92). Raloxifene hydrochloride is the first SERM used for the treatment of osteoporosis (94), with data from the MORE study showing that it increases BMD in the spine and the femoral neck, whilst decreasing the vertebral fracture risk (95). Bazedoxifene is another SERM which has been approved for the treatment of osteoporosis in post-menopausal women (96); although, it is considered inferior compared to other anti-osteoporotic drugs since it has been shown to augment the lumbar spine BMD, but not the hip BMD. Furthermore, bazedoxifene is combined with estrogens forming a tissue selective estrogen complex (TSEC), which is used to moderate vasomotor symptoms (97). Interestingly, Takamura et al. presented a case report regarding a 53-year-old woman with liver impairment and histologically confirmed NASH after the initiation of raloxifene treatment (98). Matsumura et al. reported a similar case regarding a 70-year-old woman, whose NAFLD deteriorated within three months after starting raloxifene (99). Both these clinical cases suggested that liver function should be carefully surveilled following initiation of raloxifene treatment. Contrary, the findings from Luo et al. in a choline-deficient high-fat diet NASH mouse model showed an improvement in NASH after the administration of raloxifene (100). In line with this animal study, Barrera et al. examined a recent SERM, i.e. bazedoxifene acetate (BZA), in ovariectomized female mice fed a Western diet for 10-12 weeks. In this study, BZA administration, either alone or in combination with a conjugated estrogen (CE), resulted in attenuated liver steatosis along with increases in subcutaneous and visceral white adipose tissue induced by a high-fat diet (101). Moreover, Kim et al. studied the effects of BZA and TSEC on metabolic dysfunction in ovariectomized mice fed with a high-fat diet, demonstrating that BZA and TSEC promoted hepatic lipid oxidation and improved glucose homeostasis by raising the activity of Sirtuin1 (SIRT1), PPARα and hepatic AMPK of a different mechanism of action compared to E2 and CE (102). Interestingly, a natural SERM (genistein) given as monotherapy at high doses or in combination with CE in ovariectomized mice reduced fed with a high-fat diet significantly reduced the microvesicular fat infiltration in hepatocytes and hepatic TG accumulation induced by the high-fat diet (103). In an attempt to elucidate the underlying molecular mechanism, genistein was shown to decrease the expression of peroxisome proliferator-activated receptor-gamma (*PPARγ*) which is known to play a crucial role in the progression of hepatic steatosis (103).

### 4.3 Calcitonin and NAFLD

Calcitonin is a 32 amino acids hormone which acts by inhibiting the osteoclasts and stimulating the renal calcium excretion; hence, is regarded as an anti-osteoporotic treatment (not as effective as other anti-osteoporotic drugs) (104). Although, the nasal spray of calcitonin may be used in patients that cannot tolerate other therapies, there are concerns that calcitonin may provoke malignancies, thus, it was withdrawn from the market in Europe and Canada (92). Gydesen et al. investigated the effect of a dual amylin and calcitonin receptor agonist (DACRA) on rats fed with high-fat diet, showing that this treatment resulted in improved glucose homeostasis, higher weight loss, enhanced insulin action and decreased lipid accumulation in the liver and skeletal muscles, whilst improved food preferences was also noted in these rats (105, 106). Finally, Polymeris et al. demonstrated that serum calcitonin levels increased after a 75-g oral glucose tolerance test in healthy adults, suggesting that calcitonin may be stimulated by hyperinsulinemia (107).

### 4.4 Denosumab and NAFLD

Denosumab is a human monoclonal immunoglobulin G2 antibody used as a treatment of osteoporosis. which binds to RANKL, thus inhibiting RANK activation and the formation and survival of osteoclasts. As shown by the 10-year FREEDOM Extension study, denosumab can be safely used as an anti-osteoporotic treatment for 10 years with low rates of adverse events, whilst significantly increasing the lumbar spine BMD and decreasing the incidence of fractures (108, 109). However, multiple vertebral fractures have been reported after the discontinuation of this drug, and, thus, the transition to another anti-osteoporotic therapy is important after the discontinuation of denosumab (110).

As RANK/RANKL and OPG are also expressed in other tissues (e.g. in the liver and fibroblasts), it has been suggested that the RANK/RANKL/OPG system may play a physiologic role in organs/tissues other than bone tissue (44). Indeed, the inhibition of the RANKL/RANK signaling pathway has been reported as a potential target for the treatment of T2DM and insulin resistance in humans (111). Interestingly, Zhong et al. showed that RANKL levels were gradually higher when going from control mice to high-fat diet induced NAFLD and NASH, whilst RANKL appeared to also play a role in Runx2-prompted macrophage migration (112). *In vitro*, this study also showed that Runx2 regulated the production of RANKL in hepatic stellate cells (112). Furthermore, using a mouse model that expressed human RANKL, Rinotas et al. showed that RANKL overexpression increases insulin resistance and promotes the development of NAFLD, with these effects being exerted -at least partially- by acting at a post-receptor level, as well as by upregulating the secretion of inflammatory cytokines through NFκB activation (113). Of note, the administration of denosumab appeared to reverse the negative effect of RANKL on insulin resistance (113). In line with this finding, Kiechl et al. showed in a mouse model that RANKL blockage improved hepatic insulin resistance by preventing the activation of NFκB which is known to play a role in hepatic steatosis and NAFLD (114).

Moreover, Takeno et al. presented a case report, showing NASH improvement following denosumab treatment in a woman with growth hormone deficiency and NASH (115). Observational studies have also noted an association between serum RANKL levels and NAFLD, with. Lu et al. reporting a correlation between elevated RANKL levels and higher NAFLD risk in women with PCOS (116). In addition, RANKL levels have been associated with hyperglycemia and higher T2DM risk. Recently, taking into account that increased hepatic expression of RANKL may play a role in the progression of NAFLD, Polyzos et al. proposed the use of denosumab for the treatment of NAFLD (117). However, interventional studies are required to support this suggestion.

### 4.5 Romosozumab and NAFLD

Romosozumab is a monoclonal antibody which inhibits sclerostin, an inhibitor of the Wnt signaling pathway signaling, and increases bone formation whilst reducing bone resorption. Since there are studies that have documented a relation between romosozumab treatment and cardiovascular and cerebrovascular events, it is currently recommended not to use romosozumab in patients with myocardial infarction or stroke in the last year (118).

As aforementioned, the Wnt/beta-catenin pathway appears to have an important role in the development and the progression of NAFLD (66), hence, romosozumab has also been proposed as a potential treatment for NAFLD (119). In line with this, Kim et al. using two different mouse models, namely sclerostin-deficient mice and mice treated with a sclerostin-neutralizing antibody, showed significantly increased bone mass, as well as decreased hepatic lipid accumulation and liver inflammation (120). Furthermore, Zhou et al. also revealed that sclerostin levels were reduced in NAFLD mice compared to controls (121). Finally, Oh et al. reported higher sclerostin mRNA levels in both patients with obesity and mice fed with a high-fat diet., whilst further showed that sclerostin administration amplified lipid accumulation in hepatocytes (122). On the other hand, Polyzos et al. reported decreased sclerostin levels in patients with NAFLD and NASH (123), while Rhee et al. founded that patients with advanced liver cirrhosis had higher sclerostin levels compared to healthy controls and patients with early cirrhosis (124).

Overall, the role of sclerostin and Wnt/beta-catenin in the development and progression of NAFLD appears to be complex and further research on the potential clinical impact of romosozumab on NAFLD is required to elucidate the role of this anti-osteoporotic treatment in the context of NAFLD/NASH.

### 4.6 Teriparatide and NAFLD

Teriparatide [rhPTH(1-34), the bioactive portion (1-34) of endogenous human PTH] is an anti-osteoporotic/osteoanabolic treatment (89). Feng et al. in their recent study in animal models of NAFLD using intermittent PTH administration, showed an amelioration of hepatic steatosis. They demonstrated, using an *in vitro* model of hepatic steatosis, that PTH through its receptor, induces in hepatocytes the expression of genes involved in β-oxidation and reduces the expression of genes involved in lipid uptake and *de novo* lipogenesis (125).

A recent metanalysis of 10 studies by Jaroenlapnopparat et al. demonstrated that high PTH levels was correlated with NAFLD, and their relation was statistically important. They also showed an association between PTH level and NASH, which was not statistically important (126).

## 5 Perspectives and conclusion

NAFLD and osteoporosis are highly prevalent diseases which frequently co-exist with increasing incidence globally. Although common molecular pathogenetic mechanisms/pathways (e.g. the RANKL-OPG-RANK pathway and Wnt pathway) are supported by emerging data, not all epidemiological studies point towards a positive link between these two chronic diseases. To date, a limited number studies demonstrated an associative relationship between NAFLD and osteoporosis; however, conclusive evidence for causative link(s) and their direction are still missing. Further research, both basic/translational and clinical aiming to elucidate the interplay between the liver and bones is essential, including large prospective cohort and interventional studies which could target specific patient populations with NAFLD and osteoporosis.

In this context, recent studies have been further linking sarcopenia with both NAFLD and osteoporosis, thus highlighting sarcopenia as a potential mediating factor between these two diseases. This is also supported by the fact that molecules causatively implicated in sarcopenia, such as sclerostin, RANKL, and 25(OH)-vitamin-D, already constitute therapeutic targets in osteoporosis, whilst are also considered to play a role in the pathophysiology of NAFLD (122–124). Similarly, other therapeutic targets for osteoporosis, such as kathepsin K, also seem to be implicated in NAFLD progression (127, 128). Thus, it can be proposed that there is scope to focus future research in this field among patients with coexisting NAFLD, osteoporosis and sarcopenia since this group may benefit from anti-osteoporotic drugs involved in the overlapping pathophysiological mechanisms underlying these conditions. Since no globally approved pharmacological treatment for NAFLD is available yet, whilst there is an arsenal of approved anti-osteoporotic medication, observational data as well as interventional studies could evaluate the potential impact of these anti-osteoporotic drugs on NAFLD (**Figure 1**), with focus on certain phenotypic characteristics of the patient population (e.g. sarcopenic or not). Such targeted studies may shed light in the complex and yet not fully clarified links that form the liver-bone axis.

## Author contributions

MC: Investigation, Writing – original draft. IK: Writing – review and editing. TA: Methodology, Writing – original draft. C-MF: Writing – original draft. EM: Writing – original draft. KC: Writing – original draft. YA: Writing – original draft. AP: Writing – original draft. EK: Conceptualization, Investigation, Methodology, Supervision, Writing – original draft, Writing – review and editing. HR: Investigation, Methodology, Supervision, Writing – original draft, Writing – review and editing.
